# Short-Term Effects of Phenobarbitone on Electrographic Seizures in Neonates

**DOI:** 10.1159/000443782

**Published:** 2016-03-31

**Authors:** Evonne Low, Nathan J. Stevenson, Sean R. Mathieson, Vicki Livingstone, Anthony C. Ryan, Janet M. Rennie, Geraldine B. Boylan

**Affiliations:** ^a^Neonatal Brain Research Group, Irish Centre for Fetal and Neonatal Translational Research, Department of Paediatrics and Child Health, University College Cork, Cork University Maternity Hospital, Cork, Ireland; ^b^Academic Research Department of Neonatology, Institute for Women's Health, University College London, London, UK

**Keywords:** Anti-seizure drug, Electrographic seizure burden, Multi-channel EEG, Treatment

## Abstract

**Background:**

Phenobarbitone is the most common first-line anti-seizure drug and is effective in approximately 50% of all neonatal seizures.

**Objective:**

To describe the response of electrographic seizures to the administration of intravenous phenobarbitone in neonates using seizure burden analysis techniques.

**Methods:**

Multi-channel conventional EEG, reviewed by experts, was used to determine the electrographic seizure burden in hourly epochs. The maximum seizure burden evaluated 1 h before each phenobarbitone dose (T_-1_) was compared to seizure burden in periods of increasing duration after each phenobarbitone dose had been administered (T_+1_, T_+2_ to seizure offset). Differences were analysed using linear mixed models and summarized as means and 95% CI.

**Results:**

Nineteen neonates had electrographic seizures and met the inclusion criteria for the study. Thirty-one doses were studied. The maximum seizure burden was significantly reduced 1 h after the administration of phenobarbitone (T_+1_) [-14.0 min/h (95% CI: −19.6, −8.5); p < 0.001]. The percentage reduction was 74% (IQR: 36-100). This reduction was temporary and not significant within 4 h of administrating phenobarbitone. Subgroup analysis showed that only phenobarbitone doses at 20 mg/kg resulted in a significant reduction in the maximum seizure burden from T_-1_ to T_+1_ (p = 0.002).

**Conclusions:**

Phenobarbitone significantly reduced seizures within 1 h of administration as assessed with continuous multi-channel EEG monitoring in neonates. The reduction was not permanent and seizures were likely to return within 4 h of treatment.

## Introduction

Seizures are harmful to the developing neonatal brain [1], are a neurological emergency and require prompt treatment with an anti-seizure drug (ASD). In 2011, published management guidelines for neonatal seizure by the World Health Organization strongly recommended only the use of phenobarbitone as a first-line ASD; however, it was acknowledged that this recommendation was based on very-low-quality evidence [2]. To date, phenobarbitone remains the most common first-line ASD for treatment of neonatal seizures; this practice is largely based on tradition, local protocols or personal preference as phenobarbitone has been shown to abolish seizures in only 50% of cases [3,4]. As a result, the treatment of neonatal seizures has not changed significantly in the last 50 years, although a number of potential new treatments are being investigated [5].

The development of ASDs for neonates remains a challenging area due to developmental differences between the neonatal and adult brain such as higher concentrations of intracellular chloride and a lower expression of γ-aminobutyric acid (GABA) receptors in the developing neonatal brain [6]. Evidence on the effectiveness of ASDs for neonates is translated from studies in older children and animal models. There is also inconsistency on the measurement of effectiveness of ASDs. Effectiveness assessed by clinical observation is known to be inaccurate [7,8], whilst others have used the amplitude-integrated EEG [9] which has limitations for seizure detection in neonates [10]. Effectiveness is often defined as a binary variable (effective vs. ineffective) without more detailed quantification of the actual reduction of electrographic seizures [9,11] or the duration of the effect. The complete response of electrographic seizures to individual doses of ASDs, therefore, remains poorly understood in neonates. We aimed to measure the effectiveness of individual phenobarbitone doses for the reduction of seizures using multi-channel EEG recordings and detailed seizure burden analysis in a cohort of term neonates with mixed seizure aetiology.

## Methods

As part of an ongoing study of neonatal seizures, neonates were enrolled from the neonatal intensive care units in Cork University Maternity Hospital (Ireland) and University College London Hospital (United Kingdom) from January 2009 to October 2011. Neonates ≥37 weeks’ gestation were enrolled for EEG monitoring if there was any evidence of encephalopathy or seizures within 72 h of age. Neonates who had at least one ASD dose administered during electrographic seizures were included in the study. Neonates were excluded if all their phenobarbitone doses were administered without electrographic evidence of seizures. Institutional review board approval was obtained from the Clinical Research Ethics Committees of the Cork Teaching Hospitals (Ireland) and the National Health Service in the United Kingdom via the Integrated Research Application Service. Written, informed consent was obtained from at least one parent of each neonate who participated in this study.

All clinical seizures and EEG seizures recognized by the clinical team were treated. The standardized protocol for ASD usage was similar in both hospitals. At the discretion of the attending neonatologist, a phenobarbitone loading dose of 10 or 20 mg/kg was administered intravenously on seizure recognition. Subsequent phenobarbitone doses up to an accumulated dosage of 40 mg/kg or second- (intravenous phenytoin or midazolam), third- and fourth-line ASDs were administered if clinical and/or electrographic seizures recurred. The time, dose and accumulated dosage of phenobarbitone were recorded. We assumed a zero clearance rate when calculating the accumulated dosage as the majority of doses were given well within the half-life of phenobarbitone [9,11,12]. At both hospitals, EEG recording methods were identical. A Nicolet monitor (CareFusion NeuroCare, Middleton, Wis., USA) was used to record multi-channel video-EEG using the 10-20 system of electrode placement modified for neonates [13]. The entire EEG recording from each neonate was independently reviewed by two experienced neonatal electroencephalographers (G.B.B., S.R.M.) who were blinded to clinical details. Each electrographic seizure annotated was defined according to Clancy [14] and status epilepticus was defined as by Ortibus et al. [15].

**Fig. 1:**
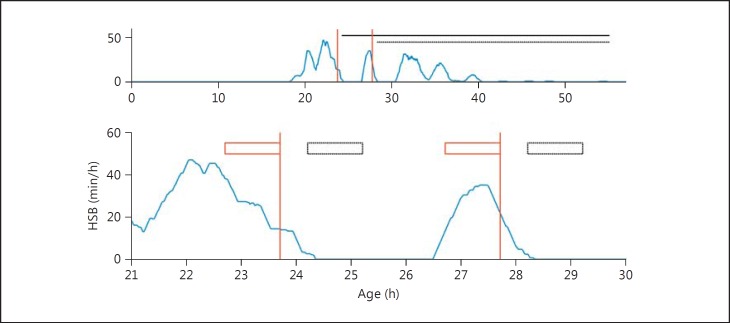
An example plot of the hourly seizure burden (HSB) over time (blue line; color in online version only) for one neonate with seizures overlaid with the time periods used to assess the effectiveness of phenobarbitone. The change in HSB was compared 1 h before (T_-1_), 1 h after (T_+1_) and in the remaining hours of electrographic recorded seizures after the administration of phenobarbitone (T_+LR_). The upper plot is the complete seizure time course for the neonate with T_+LR_ (black horizontal lines) shown for each 20 mg/kg dose of phenobarbitone (red vertical lines). After the second dose of phenobarbitone was given, electroclinical dissociation of seizures occurred and subsequent seizures were not highlighted to the clinical team, so there was no additional anti-seizure drug (ASD) given for seizures between 30 and 40 h. The lower plot is the magnified version of the upper plot with T_-1_ (red boxes) and T_+1_ (black boxes) shown for each dose of phenobarbitone (red vertical lines). There is a clear reduction in maximum HSB between T_-1_ and T_+1_ following the administration of each dose of phenobarbitone and these seizures return within T_+LR_. Note that some smoothing is apparent in the HSB as both future and past values are used to estimate the HSB and a 30-min delay is taken into account for phenobarbitone infusion.

We calculated the hourly seizure burden (HSB) in each 1-hour period of the EEG recording based on the electrographic seizure annotations. This was defined as the accumulated seizure duration within a 1-hour window, shifted across the EEG monitoring period with a 1-min interval (fig. 1). In each neonate, the maximum HSB (MSB) was used to assess the effectiveness of phenobarbitone. The MSB in a time period 1 h before a phenobarbitone dose (T_-1_) was compared to a time period of 1 h in duration beginning immediately after cessation of the phenobarbitone infusion which was completed in 30 min. The MSB in time periods of increasing duration was also used to assess the duration of the effect of phenobarbitone administration. Time periods were increased from 1 h (T_+1_), in hourly increments (T_+2_, T_+3_, T_+4_) until the last electrographically recorded seizure (T_+LR_). For example, T_-1_ is the time period from 1 h before the start of phenobarbitone infusion until the start of phenobarbitone infusion, and T_+3_ is the time period from after the cessation of phenobarbitone infusion until 3 h after the cessation of phenobarbitone infusion (fig. 1). Furthermore, the seizure burden between seizure onset and phenobarbitone administration was compared between doses which showed a complete (MSB = 0) or incomplete (MSB >0) effect.

### Statistics

Continuous variables were described using medians [interquartile ranges (IQR)] and categorical variables using frequencies. Differences between MSB before and after phenobarbitone administration were calculated for each period (T_+1_, T_+2_ until T_+LR_). Linear mixed models with a neonate-level random effect were used to account for possible correlations among observations from the same neonate (more than one phenobarbitone dose per neonate). For comparisons between groups, group was included as a fixed effect in the linear mixed model. Results based on linear mixed models were presented as means (95% CI). The comparison between MSB before and after 1 h (T_-1_ vs. T_+1_) was also performed in subgroups defined by dosage and accumulated dosage. We denote the number of neonates as n_n_ and the number of doses as n_d_. All statistical analyses were performed in SAS 9.3 (SAS Institute Inc., Cary, N.C., USA), and p < 0.05 was considered as statistically significant.

### Statement of Ethics

The parents of babies gave their informed consent and the study protocol was approved by the institute's committee on human research. We confirm that we have read the position of *Neonatology* on issues involved in ethical publication and affirm that this report is consistent with those guidelines.

## Results

During the study period, of the 35 neonates with electrographic seizures identified, 16 did not meet the inclusion criteria for ASD analysis (2 neonates received no ASD and 14 were treated before EEG monitoring commenced or when there were no accompanying electrographic seizures; for more details see supplementary material at www.karger.com/doi/10.1159/000443782). Therefore, the effectiveness of phenobarbitone was measured in the remaining 19 neonates; table 1 lists their clinical characteristics and details of seizure burden. EEG monitoring began at the median (IQR) age of 17 h (4-36), EEG duration was 78 h (56-109) and the age of first electrographic seizure was 18 h (11-41).

The 19 neonates received a total of 37 loading phenobarbitone doses during EEG monitoring, 31 of which were given during electrographic seizures (see table S1 and fig. S1 in the supplementary material at www.karger.com/doi/10.1159/000443782). The median (IQR) time between seizure onset and phenobarbitone administration was 3.3 h (1.1-10.7; n_d_ = 31). A significant MSB reduction was seen in the hour immediately after phenobarbitone administration [mean difference (95% CI): −14.0 min/h (-19.6, −8.5); p < 0.001 (n_n_ = 19; n_d_ = 31); table 2]. The median (IQR) percentage reduction was 74.0% (36.0-100.0) in the 31 doses. In 13 of the 19 neonates, a complete abolition of electrographic seizures was seen in the first hour following a loading phenobarbitone dose at 20 mg/kg. This abolition was permanent in 3 neonates. The MSB did not show a significant reduction over the longer term when comparing MSB in T_-1_ to T_+LR_ [mean difference (95% CI): −2.3 min/h (-9.2, 4.5); p = 0.481]. In fact, the MSB was not significantly reduced after phenobarbitone by T_+4_ (table 3). The seizure burden before phenobarbitone administration (20 mg/kg only) was significantly lower for doses which resulted in a complete MSB reduction in the first hour (T_+1_) [mean (95% CI): 28.1 min (-5.9, 62.1); n_d_ = 13] compared to doses which did not completely reduce the MSB [mean (95% CI): 117.6 min (71.3, 164.0); n_d_ = 7; p = 0.004]. Ten of the 13 doses which resulted in complete seizure reduction in T_+1_ were a first dose (table 2). The median (IQR) time between electrographic seizure onset and first analysed dose was 1.8 h (0.7-2.4).

**Fig. 2:**
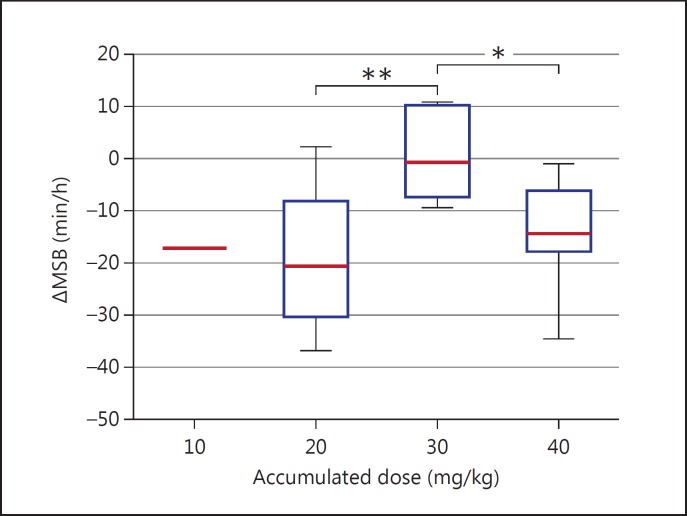
The short-term reduction in seizure burden of phenobarbitone associated with accumulated dosage. ΔMSB denotes the change in MSB between time periods T_+1_ and T_-1_. A negative ΔMSB implies a reduction in seizure burden between T_-1_ and T_+1_. The ΔMSB at accumulated doses of 20 and 40 mg/kg are significantly lower than accumulated doses of 30 mg/kg. Only 1 neonate had a dose of 10 mg/kg as a first dose (accumulated dosage of 10 mg/kg).

The MSB reduction was greater when 20 mg/kg was administered compared to a dose of 10 mg/kg [mean difference (95% CI) of 20 mg/kg (n_d_ = 20) vs. 10 mg/kg (n_d_ = 11): −18.6 min/h (-23.7, −13.5) vs. −4.4 min/h (-11.3, 2.5); p = 0.002]. In fact, 20 of 20 (100%) doses of phenobarbitone at 20 mg/kg resulted in a reduction in MSB during T_+1_, and in 13 of 20 (65%) doses the MSB was zero during T_+1_. This result is reflected when observing the effect of accumulated dosage, as the MSB reduction in T_+1_ was significantly higher for accumulated doses of 20 mg/kg [mean difference (95% CI): −19.0 min/h (-25.0, −12.9); n_d_ = 14] and 40 mg/kg [mean difference (95% CI): −14.3 min/h (-21.3, −7.4); n_d_ = 10] compared to an accumulated dose of 30 mg/kg [mean difference (95% CI): −1.0 min/h (-10.0, 8.0) (n_d_ = 6); p = 0.001 and 0.017, respectively (fig. 2)].

A total of 13 doses of additional ASDs were given to neonates who received phenobarbitone as a first-line ASD (phenytoin, midazolam, clonazepam). Only 1 dose of phenobarbitone at 20 mg/kg was administered after the administration of second-line ASD (<1 h after phenytoin). No second- or third-line ASDs were given in T_+1_. The median time between phenobarbitone administration and additional lines of ASD administration was 8.3 h (5.2-12.4). Additional details on these ASDs can be found in the supplementary material at www.karger.com/doi/10.1159/000443782.

## Discussion

We have shown that a loading dose of phenobarbitone results in an immediate but temporary reduction in electrographic seizure burden in most term neonates; seizures returned to pre-treatment levels within 4 h of administration. We have also shown that 20 mg/kg doses were more effective than 10 mg/kg, and that phenobarbitone was more likely to abolish seizures in the short term if given before a large accumulation of seizures was apparent.

Phenobarbitone, a barbiturate, primarily has an inhibitory effect in the adult brain by prolonging the action of GABA, acting mainly on the GABA_A_ receptors [6]. The purported effect of phenobarbitone (seizure cessation via GABA agonism) is somewhat problematic given the large body of evidence suggesting that GABA is excitatory in the neonatal brain [16,17]. This excitatory drive may be due to the predominance of the sodium-potassium-chloride cotransporter isoform 1, which moves chloride into the cell, and the lower expression of potassium-chloride cotransporter isoform 2, which moves chloride out of the cell [6]. This suggests, and has been shown in animal models, that a GABA agonist will facilitate seizures in the developing neonatal brain. However, GABA antagonists do not reduce seizures [18] and phenobarbitone abolishes seizures in 50% of cases [3,4]. We have shown that phenobarbitone abolishes seizures during T_+1_ in 65% of cases when the dose is 20 mg/kg. Conflicting results between animal and clinical studies must be resolved in order to develop improved treatment strategies for neonatal seizures.

We have shown that the effectiveness of phenobarbitone is temporary and the reduction in seizure burden is limited beyond 4 h of administration. This is conspicuously shorter than the pharmacokinetic half-life of phenobarbitone (range: 45-500 h [12] and is variable depending on circumstances [9,11]). Phenobarbitone resistance can occur in the neonatal brain and a change in the GABA_A_ receptor subunit, activation of a non-functional ‘spare’ GABA_A_ receptor, uncoupling of receptors and post-translational modification of GABA_A_ receptors have been hypothesized as possible mechanisms for this pharmacoresistance [19]. We have also shown that doses of phenobarbitone were more likely to abolish seizures in the short term if they were given before a large accumulation of seizures has occurred. This implies that rapid identification of seizures and intervention, when the accumulated seizure duration is low, may be more beneficial. Nardou et al. [20] hypothesized that the increase in intracellular chloride levels during recurrent seizures may enhance the excitatory component of GABA, causing GABA agonists such as phenobarbitone to be ineffective in reducing seizure burden.

Seizures are intermittent and highly variable, and show a natural tendency to decay after a long period of time [21]. Up to 80% of neonatal seizures may be missed using clinical observation alone; methods such as the amplitude-integrated EEG are unreliable and dissociation of electroclinical seizures increases after ASD usage [7,8,10,14,22]. It is not surprising that many seizures were treated before monitoring began or when no electrographic seizures were evident.

In maternity units like ours, which monitor neonates with seizures intensively, we still found suboptimal treatment of neonatal seizures. If electrographic seizures emerged during out-of-hours periods, there were no alarm systems to alert clinical teams to ongoing electrographic seizures [23], hence treatment was not always instigated promptly. We are aware that this is a heterogeneous group, and while the numbers (n = 19) in our study were sufficient for the general assessment of phenobarbitone effectiveness on neonatal seizures, it was insufficient for assessment of phenobarbitone effectiveness with respect to seizure aetiology, dosing strategies or therapeutic hypothermia. The presence of second- and third-line ASDs would have resulted in a possible underestimation of the MSB in longer-duration post-phenobarbitone time periods. This, however, would not change our conclusion on the short-term effect of phenobarbitone, as only 1 dose of phenobarbitone was given in the presence of a second-line ASD. In fact, an underestimation of MSB only enhances our findings relating to a short-term reduction in seizures and that seizure re-occurrence is earlier than can be accounted for by biological clearance.

## Conclusion

Phenobarbitone immediately reduced the accumulation of seizures in a cohort of neonates with mixed aetiology. The effect was temporary and the reduction in seizure burden was not significant within 4 h of treatment. Doses of phenobarbitone at 20 mg/kg, rather than 10 mg/kg, were significantly more effective in reducing seizure burden. Phenobarbitone was also more effective if administered when the seizure burden was relatively low.

## Disclosure Statement

None of the authors has any conflict of interest to disclose.

## Figures and Tables

**Table 1:** Summary characteristics of the 19 neonates included for the study analysis

Age at EEG monitoring, h	17 (4–36)
Age of first EEG seizure, h	18 (l1–41)
Duration of EEG monitoring, h	78 (56–109)
Summary of seizure burden	
Recorded seizure burden, min	119 (45–305)
Seizure number	25 (11–13o)
Mean seizure duration, s	183 (126–298)
Neonates with status epilepticus	9
Neonates who received therapeutic	
hypothermia	7
Age of first ASD, h	19 (11–51)

*Clinical diagnosis*	
HIE grade II	5 (3 were cooled)
HIE grade III	5 (4 were cooled)
Multiple infarction	2
Focal arterial infarction	2
Bifocal arterial infarction	2
Suspected viral encephalitis	1
Unknown cause	1
Benign non-familial seizures	1

Data are expressed as n or medians (IQR). HIE = Hypoxicischaemic encephalopathy; ASD = anti-seizure drug.

**Table 2:** Details on the administration of phenobarbitone and the maximum hourly seizure burden (MSB) in the 19 neonates with 31 doses of phenobarbitone analysed

Case	PB dose in sequence, mg/kg	PB dose analysed, mg/kg	Accumulated dose, mg/kg	MSB T_-1_, min/h	MSB T_+1_, min/h	% reduction Between T_+1_ and T__1_	MSB, min/h											
							T_+2_	T_+3_	T_+4_	T_+5_	T_+6_	T_+7_	T_+8_	T_+9_	T_+10_	T_+11_	T_+12_	^T^_+LR_
1	20^a^, 10	10^2nd^ dose	30	13.9	4.5	67.5	5.9	5.9	7.4	7.4	7.4	7.4	7.4	7.4	7.4	7.4	7.4	7.4
2	20	20^1st^ dose	20	34.2	0.0	100.0	0.0	0.0	0.0	0.0	0.0	0.0	0.0	0.0	0.0	0.0	0.0	0.0
3	20^a^, 10	10^2nd^ dose	30	8.9	12.0	– 35.4	12.0	12.0	12.0	12.0	12.0	12.0	12.0	12.0	12.0	12.0	12.0	12.0
4	20	20^1st^ dose	20	30.2	0.0	100.0	2.4	2.4	14.5	14.5	14.5	14.5	14.5	14.5	14.5	14.5	14.5	14.6
5	20	20^1st^ dose	20	8.1	0.0	100.0	0.0	0.0	0.0	0.0	0.0	0.0	0.0	0.0	0.0	0.0	0.0	0.0
6	20	20^1st^ dose	20	36.8	0.0	100.0	0.0	0.0	0.0	0.0	0.0	0.0	0.0	0.0	0.0	0.0	0.0	0.0
7	20	20^1st^ dose	20	29.0	7.6	74.0	31.7	37.5	37.5	37.5	37.5	37.5	37.5	37.5	37.5	37.5	37.5	37.5
7	*20, 20*	20^2nd^ dose	40	34.6	0.0	100.0	0.0	0.0	0.0	3.4	27.0	27.4	27.4	27.4	27.4	27.4	27.4	27.4
8	*20, 10,* 10	20^1st^ dose	20	14.6	0.0	100.0	0.0	0.0	0.0	0.0	0.0	0.0	0.0	0.0	0.0	0.0	0.0	23.9
8	20, *10, 10*	10^2nd^ dose	30	7.2	17.4	–141.7	21.0	22.9	23.3	23.9	23.9	23.9	23.9	23.9	23.9	23.9	23.9	23.9
8	20, 10, 10	10^3rd^ dose	40	22.8	8.4	63.0	17.1	18.2	18.3	19.3	19.3	19.3	19.3	19.3	19.3	19.3	19.3	19.3
9	20^a^, 20	20^2nd^ dose	40	7.2	0.0	100.0	0.0	0.0	0.0	7.5	7.5	7.5	7.5	7.5	7.5	7.5	7.5	7.5
10	*20, 10*^a^*, 10*	20^1st^ dose	20	17.5	0.0	100.0	0.0	8.9	23.9	25.4	27.4	57.9	58.7	60.0	60.0	60.0	60.0	60.0
10	20, 10^a^, 10	10^3rd^ dose	40	60.0	42.2	29.6	60.0	60.0	60.0	60.0	60.0	60.0	60.0	60.0	60.0	60.0	60.0	60.0
11	*10, 10,* 20	10^1st^ dose	10	26.0	8.8	66.0	11.0	12.2	12.2	12.2	12.2	12.2	12.2	12.2	12.2	12.2	12.2	12.2
11	10, *10, 20*	10^2nd^ dose	20	9.9	12.2	– 23.4	12.2	12.2	12.2	12.2	12.2	12.2	12.2	12.2	12.2	12.2	12.2	12.2
11	10, 10, 20	20^3rd^ dose	40	2.5	1.5	40.3	7.0	7.8	7.8	7.8	7.8	7.8	7.8	7.8	7.8	7.8	7.8	7.8
12	*20, 10*	20^1st^ dose	20	3.4	0.0	100.0	7.7	11.0	11.0	11.0	11.0	11.0	11.0	11.0	11.1	11.1	11.1	23.3
12	20, 10	10^2nd^ dose	30	11.1	6.7	39.9	15.6	23.3	23.3	23.3	23.3	23.3	23.3	23.3	23.3	23.3	23.3	23.3
13	*20, 20*	20^1st^ dose	20	29.4	0.0	100.0	4.7	9.1	16.7	16.7	16.7	16.7	16.7	16.7	16.7	16.7	16.7	16.7
13	20, 20	20^2nd^ dose	40	16.7	2.3	86.4	2.3	3.4	7.0	7.0	7.0	7.0	7.0	7.0	7.0	7.0	7.0	7.0
14	*20, 20*	20^1st^ dose	20	47.7	14.7	69.3	19.8	25.0	27.2	29.0	31.9	36.2	36.2	36.2	36.2	36.2	36.2	36.2
14	20, 20	20^2nd^ dose	40	35.0	20.8	40.5	25.2	25.9	26.5	26.5	26.5	26.5	26.5	26.5	26.5	26.5	26.5	26.5
15	20^a^, *10, 10*	20^2nd^ dose	30	30.6	41.3	– 35.2	41.3	43.2	43.2	43.2	43.2	43.2	43.2	43.2	43.2	43.2	43.2	43.2
15	20^a^, 10, 10	10^3rd^ dose	40	39.8	35.1	12.0	42.6	42.6	42.6	42.6	42.6	42.6	42.6	42.6	42.6	42.6	42.6	42.6
16	*20, 10*	20^1st^ dose	20	23.3	0.7	96.9	16.0	27.7	27.7	27.7	27.7	27.7	27.7	27.7	27.7	27.7	27.7	27.7
16	20, 10	20^2nd^ dose	30	20.1	12.9	36.0	12.9	12.9	12.9	12.9	12.9	12.9	12.9	12.9	12.9	12.9	12.9	12.9
17	20^a^, 20	20^2nd^ dose	40	20.9	0.0	100.0	0.0	0.0	0.0	0.0	0.0	0.0	0.0	6.0	6.0	6.0	6.0	7.7
18	*20, 20*	20^1st^ dose	20	19.8	0.0	100.0	15.2	15.2	22.1	22.1	22.1	22.1	27.8	27.8	27.8	27.8	27.8	31.9
18	20, 20	20^2nd^ dose	40	21.9	15.8	27.8	18.6	20.9	31.9	31.9	31.9	31.9	31.9	31.9	31.9	31.9	31.9	31.9
19	20	20^1st^ dose	20	1.7	0.0	100.0	0.0	0.0	0.0	0.0	0.0	0.0	0.0	0.0	0.0	0.0	0.2	0.9

The first dose (n = 14), second dose (n = 13) and third dose (n = 4) of phenobarbitone were analysed. Italics: time calculated between phenobarbitone doses analysed. The change in MSB is compared 1 h before (T_–1_), 1 h after (T_+1_) and in hourly increments in the remaining hours after the administration of phenobarbitone (T_+LR_). PB = Phenobarbitone.

a Phenobarbitone dose given but before EEG monitoring began or not during electrographic seizures.

**Table 3:** Results of linear mixed models for the reduction in maximum hourly seizure burden between T_–1_ (1 h before phenobarbitone) and T_+1_ (1 h after phenobarbitone) from 31 observations at each time point across the 19 neonates

MSB: Post-phenobarbitone administration	Mean difference (95% CI), min/h	p values
Post: 1 h	−14.04 (–19.60 to −8.48)	<0.001
Post: 2 h	−9.48 (–15.05 to −3.91)	0.003
Post: 3 h	−7.53 (−13.34 to −1.73)	0.016
*Post: 4 h*	−*5.38 (−11.15 to 0.39)*	*0.064*
Post: 5 h	−4.89 (−10.70 to 0.93)	0.089
Post: 6 h	−4.99 (−10.88 to 0.91)	0.090
Post: 7 h	−3.39 (−9.78 to 2.99)	0.268
Post: 8 h	−3.29 (−9.78 to 3.21)	0.292
Post: 9, 10, 11 h	−2.92 (−9.31 to 3.46)	0.338
Post: 12 h	−2.92 (−9.31 to 3.47)	0.338
Until T_+LR_	−2.33 (−9.20 to 4.54)	0.481

Values in italics represent the time at which p > 0.05.
